# Supplementary material for: Global hypo-methylation in a proportion of glioblastoma enriched for an astrocytic signature is associated with increased invasion and altered immune landscape
Source: eLife. 2022 Nov 22;11:e77335. doi: 10.7554/eLife.77335 (PMC9681209; doi:10.7554/eLife.77335)
Supplement: Figure 2—source data 1. [file elife-77335-fig2-data1.zip › Figure_2_source_data_1/Figure_2B/homerResults/motif8.similar.html]

motif8

## Information for motif8

A
C
G
T
A
C
T
G
A
C
T
G
A
G
T
C
A
G
T
C
T
G
A
C
A
C
G
T
A
C
T
G
  
Reverse Opposite:  

A
G
T
C
C
G
T
A
A
C
T
G
A
C
T
G
A
C
T
G
A
G
T
C
T
G
A
C
C
G
T
A
  

|  |  |
| --- | --- |
| p-value: | 1e-70 |
| log p-value: | -1.630e+02 |
| Information Content per bp: | 1.923 |
| Number of Target Sequences with motif | 160.0 |
| Percentage of Target Sequences with motif | 6.92% |
| Number of Background Sequences with motif | 4.5 |
| Percentage of Background Sequences with motif | 1.27% |
| Average Position of motif in Targets | 98.9 +/- 54.3bp |
| Average Position of motif in Background | 96.5 +/- 55.7bp |
| Strand Bias (log2 ratio + to - strand density) | -0.3 |
| Multiplicity (# of sites on avg that occur together) | 1.06 |
| Motif File: | file (matrix) reverse opposite |

### Similar de novo motifs found

|  |  |  |  |  |  |  |  |
| --- | --- | --- | --- | --- | --- | --- | --- |
| Rank | Match Score | Redundant Motif | P-value | log P-value | % of Targets | % of Background | Motif file |
| 1 | 0.698 | A G T C C G T A C T A G C T A G T A C G T A G C T G A C C T G A A T C G T A C G C G A T A C T G C T G A C T A G T C A G | 1e-62 | -143.856921 | 3.68% | 0.48% | motif file (matrix) |
| 2 | 0.700 | A C T G A C T G C G T A A C G T A C T G A G T C C G T A A C T G A C T G A C T G A G T C A G T C | 1e-57 | -131.647631 | 4.54% | 0.80% | motif file (matrix) |
| 3 | 0.619 | C G T A A T C G T A G C C G A T A C T G A G C T A T G C G A T C G T A C C A G T | 1e-40 | -92.296128 | 5.15% | 1.13% | motif file (matrix) |
| 4 | 0.617 | A G T C G A T C C G A T C A T G A T C G T G C A A C G T A T G C G C A T A T C G T A G C G T C A | 1e-36 | -83.647374 | 3.50% | 0.82% | motif file (matrix) |
| 5 | 0.675 | A G T C C G T A A G T C A C T G C T A G A G T C | 1e-32 | -74.384455 | 6.84% | 2.53% | motif file (matrix) |
| 6 | 0.608 | A T C G A C T G T C G A A G T C C G T A C G T A | 1e-24 | -55.696797 | 21.77% | 13.98% | motif file (matrix) |
